# The Duality of Adiponectin: The Role of Sex in Atherosclerosis

**DOI:** 10.3390/cells13010001

**Published:** 2023-12-19

**Authors:** Abigail E. Cullen, Ann M. Centner, Riley Deitado, Vladimir Ukhanov, Judy Muller-Delp, Gloria Salazar

**Affiliations:** 1 Department of Health, Nutrition and Food Sciences, Florida State University, Tallahassee, FL 32306, USA; acullen@uoregon.edu (A.E.C.); ann.centner@med.fsu.edu (A.M.C.); rdeitado@fsu.edu (R.D.); vukhanov@fsu.edu (V.U.); 2 Department of Human Physiology, University of Oregon, Eugene, OR 97403, USA; 3 Department of Biological Sciences, Florida State University, Tallahassee, FL 32306, USA; judy.delp@med.fsu.edu; 4 Center for Advancing Exercise and Nutrition Research on Aging (CAENRA), Florida State University, Tallahassee, FL 32306, USA

**Keywords:** atherosclerosis, adiponectin, sex, MCP1

## Abstract

The hormone adiponectin has many beneficial effects in atherosclerosis, as gene deficiency in adiponectin or its receptor has shown detrimental effects on plaque burden in mice. Our objective was to understand the potential roles adiponectin deficiency has on aortic plaque content, inflammation, and markers of cardiovascular disease according to sex and age. To study the influence of adiponectin status on sex and atherosclerosis, we used young male and female *adipoq*^−/−^ *apoe*^−/−^, *adipoq^+/^*^−^*apoe*^−/−^, and *apoe*^−/−^ mice, which were given a high-fat diet (HFD). Even a 50% reduction in the expression of adiponectin led to a plaque reduction in males and an increase in females compared with *apoe*^−/−^ controls. Changes in plaque were not attributed to changes in cholesterol or cardiovascular disease markers but correlated with inflammatory markers. Plaque reduction in males was associated with reduced monocyte chemoattractant protein 1 (MCP1) and increased colony stimulating factor 3 (CSF3), while the increase in plaque in females correlated with the opposite effect in these markers. In old mice, both adiponectin-deficient genotypes and sexes accumulated more plaque than their respective *apoe*^−/−^ controls. The increase in plaque with adiponectin deficiency according to age was not explained by a worsening lipid profile but correlated with increased levels of C-C motif chemokine ligand 5 (CCL5). Overall, our study uncovered genotype-specific effects that differed by sex and age of adiponectin deficiency in atherosclerosis.

## 1. Introduction

Atherosclerosis, the major cause of cardiovascular diseases (CVDs), is exacerbated by risk factors including aging, obesity, and dietary choices. An additional risk factor arising from obesity and aging is the reduction of adiponectin [1,2]. Adiponectin, a hormone secreted in high levels by adipocytes, has been shown to positively alter the phenotype of vascular smooth muscle cells (VSMCs) [3]; thus, the decreased circulating levels of adiponectin in disease states put the body at heightened risk for other CVDs [4]. Although the correlation between hypoadiponectinemia and atherosclerosis has been shown, specific mechanisms behind its role in mitigating plaque formation have yet to be elucidated [5,6].

Adiponectin is secreted mainly by adipose tissue; however, other tissues are also sources of this hormone, including VSMCs and cardiac cells [1,3]. In VSMCs, adiponectin promotes a contractile phenotype by binding to the adiponectin receptors R1 and R2 and T-cadherin, suggesting that adiponectin may protect from atherosclerosis. However, the role of adiponectin in atherosclerosis is controversial, as treatment with adiponectin reduces atherosclerosis in male apolipoprotein E (*apoe*)^−/−^ mice [7], while lack of the adiponectin gene in low-density lipoprotein receptor (LDLR) *ldlr*^−/−^ or *apoe*^−/−^ mice showed no effect on plaque [8]. Plaque trended downwards in male *apoe*^−/−^*adipoq*^−/−^ mice. Both sexes were included for the *ldlr*^−/−^, but not for the *apoe*^−/^,^−^ background. Thus, the role of sex and the specific sites of plaque accumulation in the aortic tree in response to adiponectin deficiency need further elucidation.

In this study, we used male and female *apoe*^−/−^*adipoq*^−/−^ mice and uncovered a genotype-dependent effect on plaque accumulation that differed by sex and age. Less plaque was seen in the arch and descending aorta of young *apoe*^−/−^*adipoq*^−/−^ males treated with HFD for 5 weeks compared with *apoe*^−/−^ controls. Interestingly, the opposite was observed in female mice. The protective effect of adiponectin deficiency in males was lost during aging, as 1-year-old males and females fed a normal chow diet had more plaque compared with *apoe*^−/−^ controls. Furthermore, unlike other studies, we have shown genotype-specific effects on plaque that differed by sex as well as sex-specific differences in circulating pro-inflammatory molecules that warrant further investigation. We also show for the first time that even a 50% reduction in adiponectin achieved using the *apoe*^−/−^*adipoq^+/^*^−^ mouse has a significant impact on inflammation and the development of atherosclerosis. Furthermore, our study suggests that females with reduced adiponectin levels may be at a higher risk of developing atherosclerosis.

## 2. Materials and Methods

### 2.1. Animal Model

The animal procedures conform to the Guide for the Care and Use of Laboratory Animals and were approved by the Institutional Animal Care and Use Committee at Florida State University. The mice were housed in a 12:12 light: dark cycle with water and food provided ad libitum. Single knockout mice for Apoe (B6.129P2-Apoetm1Unc/J, Strain # 2052) and adiponectin (B6.129-Adipoqtm1Chan/J, Strain # 8195) were purchased from Jackson Laboratory and crossed to generate *apoe*^−/−^*adipoq*^−/−^ and *apoe*^−/−^*adipoq^+/^*^−^ experimental animals. Three- to four-month-old male and female mice (*n* = 8/group) were used for this study. To accelerate the formation of plaque, animals were fed a modified Paigen’s atherogenic purified HFD for 5 weeks. We have previously shown that 5 weeks of treatment with this diet induces a significant increase in plaque in male and female *apoe*^−/−^ mice [9,10,11]. The HFD contains (in % Kcal) 34.9 fat, 45.1 carbohydrates, 20.1 protein, and 0.5% cholic acid. Food and water consumption and body weights were recorded weekly. Upon completion of 5 weeks, animals were euthanized with CO_2_. Blood was collected from the left ventricle and left to coagulate, and serum was stored at −80 °C.

### 2.2. Aortic Plaque Measurement

Aortas were cleaned of periadventitial fat, fixed in 0.2% glutaraldehyde, and opened longitudinally for plaque analysis, as reported [11]. Aortas were then pinned in a petri dish containing black wax and photographed for the quantification of plaque. This measurement was performed by determining the area of plaque compared to the total area of the aorta using ImageJ software 1.53. The percent plaque was calculated for the arch and descending aorta.

### 2.3. Cytokine, Chemokines, and Lipids Measurements

The Mouse Cytokine/Chemokine Panel (MCYTOMAG-70K) was obtained from Sigma-Aldrich (St. Louis, MO, USA) to assess inflammatory markers in serum. The 96-well plate was washed with wash buffer and incubated on a shaker at room temperature (20–25 °C) for 10 min. The solution was decanted and standards (25 μL/well) or controls (25 μL/well) were added to the appropriate wells, then assay buffer (25 μL/well), matrix solution (25 μL/well), and diluted samples (25 μL/well) were added to sample wells. Lastly, magnetic beads (25 μL/well) were added. Plates were incubated for 16–20 h at 4 °C. The following day, the well contents were removed, and 2 washes with 200 μL/well wash buffer were performed. Detection antibodies (25 μL/well) were added, and the plate was incubated and covered with aluminum foil on a shaker for 1 h. Streptavidin–phycoerythrin (25 μL/well) was added, and the plate was incubated for 30 min with gentle agitation. The contents were removed and washed twice with wash buffer (200 μL/well). Sheath/drive fluid was added to each well (150 μL), and the plate was read using a MAGPIX system (Luminex Corporation, Austin, TX, USA).

For the analysis of the lipid profile, the serum was diluted 1:10 in PBS, and 100 µL of the diluted sample was added to a lipid panel cartridge (McKesson, Irving, TX, USA, 07P0205) and read in a Piccolo Xpress Metabolic analyzer (Abbott, Orlando, FL, USA).

### 2.4. Statistics

Data for plaque, lipids, and inflammatory markers were analyzed by comparing WT and *adipoq*^−/−^ and or *adipoq^+/^*^−^ mice (2 groups) using two-way *t*-tests in JMPPro15 (version 15.0.0). JMPPro15 was used for ANOVA and Tukey’s Post Hoc analysis for comparison of body weight, food intake, and water consumption for in vivo studies. For box and whisker plots, some individual values are similar, so only one dot appears on the graphs. Thus, data points on graphs are not indicative of sample size. Values are given as mean ± standard deviation of mean (SD), except for body weight, food, and water, which are given as mean ± standard error (SE), and *p* < 0.05 is considered statistically significant. Correlations were run using SPSS version 29.

## 3. Results

### 3.1. Plaque Is Reduced in Adiponectin-Deficient Males and Increased in Females

**Figure 1:**
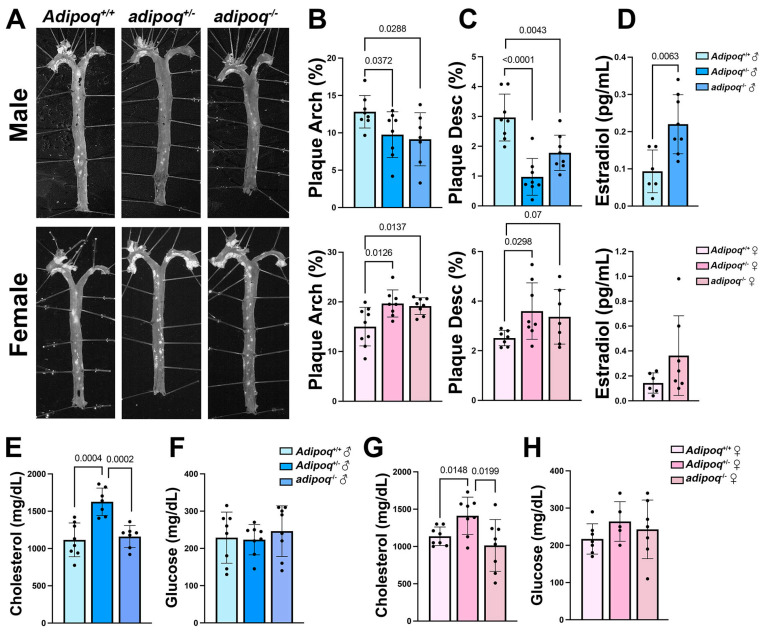
Loss of adiponectin reduces plaque in males and increases it in females. En face analysis of plaque accumulation in male and female *apoe*^−/−^*adipoq^+/+^*, *apoe*^−/−^*adipoq^+/^*^−^, and *apoe*^−/−^*adipoq*^−/−^ mice after 5 weeks of HFD treatment (*n* = 8 per group for males and 7–9 per group for females) (**A**). Plaque was quantified in the arch (**B**) and descending aorta (**C**). Serum from male and female *apoe*^−/−^*adipoq^+/+^* and *apoe*^−/−^*adipoq*^−/−^ mice was tested for estradiol (**D**). Serum from males and females in the 3 genotypes was used to measure cholesterol (**E**,**G**) and glucose (**F**,**H**).

To test the role of adiponectin in atherosclerosis, male and female *apoe*^−/−^*adipoq^+/+^, apoe*^−/−^*adipoq^+/^*^−^*,* and *apoe*^−/−^*adipoq*^−/−^ mice were fed HFD for 5 weeks ad libitum. In contrast to previous reports [8], en face analysis (Figure 1A–C) of the aortas of male *apoe*^−/−^*adipoq^+/^*^−^ and *apoe*^−/−^*adipoq*^−/−^ mice indicated a significant reduction in plaque in the arch (*p* = 0.0372 and 0.0288, respectively) and descending aorta (*p* = 6.2 × 10^−5^ and 0.0043, respectively) compared with *apoe*^−/−^ controls. Females, on the other hand, had more plaque in the arch for both genotypes (*p* = 0.0126 and 0.0137, respectively) and more plaque in the descending aorta of *apoe*^−/−^*adipoq^+/^*^−^ mice (*p* = 0.0298). A trend for higher plaque accumulation was also seen in the descending aorta of *apoe*^−/−^*adipoq*^−/−^ female mice. Due to the beneficial impact of estrogen on the cardiovascular system [12], we measured estradiol using Luminex technology (Figure 1D). We used plasma from the *apoe*^−/−^ control and *apoe*^−/−^*adipoq*^−/−^ mice for this analysis. Consistent with the reduction in plaque, estradiol was elevated in males lacking adiponectin compared with *apoe*^−/−^ controls (*p* = 0.0063). A trend toward a higher level of estrogen was seen for *apoe*^−/−^*adipoq*^−/−^ female mice.

The genotype-dependent effect of adiponectin on plaque accumulation was not correlated with circulating cholesterol or glucose. Surprisingly, cholesterol was elevated in both sexes in heterozygotes (Figure 1E,G), and glucose did not differ between groups (Figure 1F,H). Also, no major differences were observed in HFD intake, water consumption, or body weight (BW) in males (Figure S1A–C) or females (Figure S1D–F). In fact, in week 5, the heterozygous male group ate significantly more than the *apoe*^−/−^ and *apoe*^−/−^*adipoq*^−/−^ groups (*p* = 0.017 and *p* = 0.02, respectively), and in week 2, the *apoe*^−/−^*adipoq*^−/−^ group drank more water than the *apoe*^−/−^ and *apoe*^−/−^*adipoq^+/^*^−^ groups (*p* = 0.006 and 0.04, respectively). There were no significant differences in food and water consumption or body weight for the female groups fed HFD.

**Figure 2:**
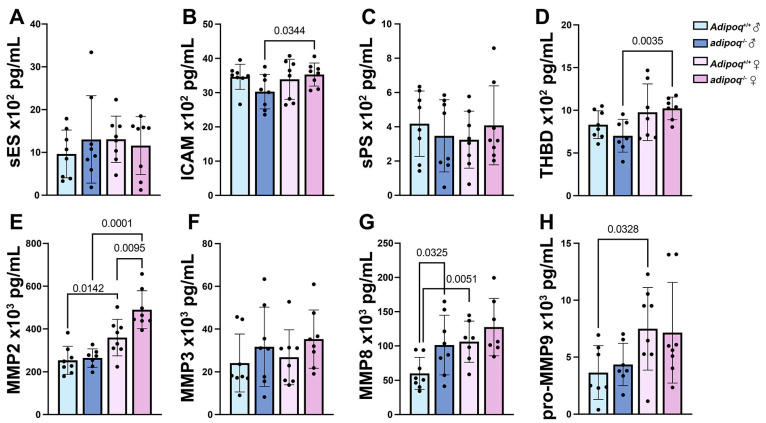
Cardiovascular disease markers do not correlate with plaque accumulation. Serum from male and female *apoe*^−/−^*adipoq^+/+^* and *apoe*^−/−^*adipoq*^−/−^ mice fed HFD for 5 weeks was tested for soluble E selectin (sES) (**A**), ICAM (**B**), soluble P selectin (sPS) (**C**), THBD (**D**), MMP2 (**E**), MMP3 (**F**), MMP8 (**G**), and pro-MMP9 (**H**).

To obtain insights into possible mechanisms, we measured CVD markers. No differences were seen between *apoe^−/−^* and *apoe^−/−^adipoq^−/−^* mice in circulating levels of soluble E and P selectins (sES, sPS), soluble intercellular adhesion molecule 1 (sICAM-1), pro-matrix metalloproteinase (MMP) 9, or thrombomodulin (THBD) in males or females (Figure 2A–D). However, sex differences were seen for ICAM and THBD, which were elevated in females compared with male *apoe^−/−^adipoq^−/−^*^.^mice. For the MMP panel, *apoe^−/−^adipoq^−/−^* males had higher levels of MMP8 (*p* = 0.0325), while females had elevated MMP2 (*p* < 0.01) compared with *apoe^−/−^* controls (Figure 2E,G). For sex differences, MMP2 (Figure 2E) was elevated in females for both genotypes compared with males, while MMP8 and pro-MMP9 were elevated in females compared with male apoe^−/−^ mice (Figure 2G,H). Thus, these results indicate that the loss of adiponectin did not promote atherosclerosis through any of these prominent pro-atherogenic pathways.

### 3.2. Adiponectin Has Sex-Dependent Effects on Inflammation

**Figure 3:**
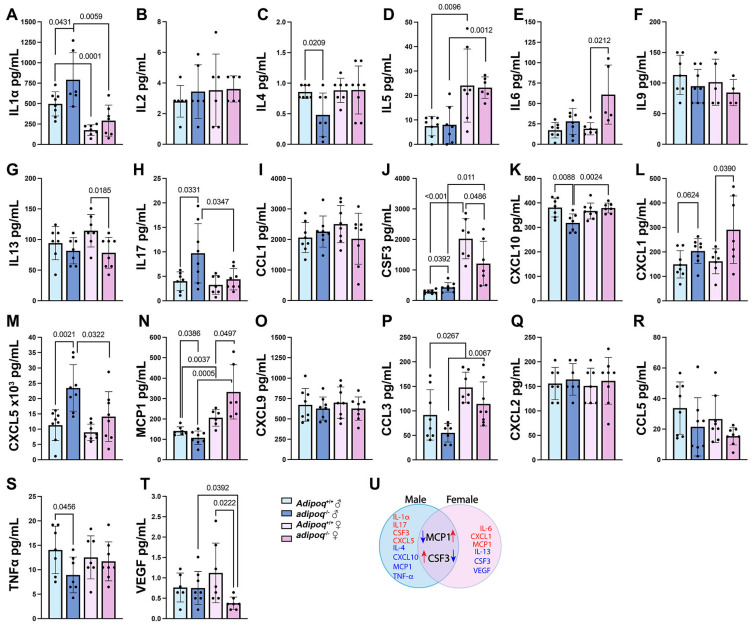
Inflammatory markers and chemokines are influenced by adiponectin genotype and sex. Serum samples from male and female *apoe^−/−^adipoq^+/+^* and *apoe^−/−^adipoq^−/−^* animals treated for 5 weeks with HFD were tested for the expression of inflammatory markers and chemokines using the Milliplex mouse cytokine/chemokine panel 1 (**A**–**T**). Overall changes are summarized in (**U**). Blue arrows indicate downregulation and red arrows indicate upregulation in expression.

To determine if the changes seen in plaque by HFD were associated with changes in inflammation, we used Luminex technology to assess the expression of pro-inflammatory cytokines and chemokines, focusing only on double knockouts (KOs) (Figure 3). There were no significant changes between sexes and genotypes for Interleukin (IL) 9, eotaxin/CC motif chemokine (CCL) 11, monokine-induced by gamma interferon (MIG)/C-X-C motif chemokine (CXCL) 9, macrophage inflammatory marker (MIP2)/CXCL2, or regulated on activation, normal T cell expressed and secreted (Rantes)/CCL5 (Figure 3F,I,O,Q,R). Changes seen in males by adiponectin deficiency included upregulation of IL1α, IL17A, granulocyte colony-stimulating factor (G-CSF)/CSF3, CXCL5, and a trend for CXCL1 (Figure 3A,H,J,M,L). A downregulation in expression was seen for IL4, CXCL10/interferon gamma-induced protein 10 (IP-10), monocyte chemoattractant protein 1 (MCP1), and tumor necrosis α (TNFα) (Figure 3C,K,N,S). In females, the lack of adiponectin upregulated IL6, keratinocyte chemoattractant (KC/CXCL1), and MCP1 (Figure 3E,L,N) and downregulated IL13, CSF3, and vascular endothelial growth factor α (VEGFα) (Figure 3G,J,T). Compared with males, females in both groups had reduced IL1α and elevated IL-5, CSF3, MCP1, and macrophage inflammatory protein-1α (MIP1α/CCL3) (Figure 3A,D,J,N,P). Altogether, we observed genotype- and sex-specific differences in inflammatory markers (Figure 3U). The downregulation of IL4, CXCL10, MCP1, TNFα, and estradiol and CSF3 upregulation may mediate the adaptation of adiponectin-deficient males to HFD-induced atherosclerosis.

### 3.3. Plaque Area in the Aortic Tree Correlates with MCP1 Expression

We investigated potential correlations between plaque accumulation within the arch of the aorta and the descending aorta and markers of cardiovascular disease, inflammation, and MMPs. First, we ran correlations between the serum markers collected and the percent area of plaque accumulation for all animals combined to uncover any correlations (Table 1). A positive, moderate correlation was seen between plaque accumulation in the arch and IL5, and strong correlations were seen between MCP1 and MMP2. Plaque accumulation in the arch was negatively correlated to IL1α and IL17. In contrast, plaque accumulation in the descending aorta was only moderately correlated to IL4 and MCP1. We assessed if correlations existed between plaque accumulation and cholesterol (CHOL), the liver enzymes alanine transaminase (ALT) and aspartate amino transferase (AST), and glucose; however, none were found.

Furthermore, we assessed if there were any correlations between sex and plaque and sex and serum markers (Figure S2). We found that there was a strong correlation between sex and plaque area in the arch, but no correlation between sex and plaque in the descending aorta. G-CSF, IL5, MIP1α/CCL3, and MMP2 showed a strong correlation to sex, whereas MMP8 only showed moderate correlation. Some markers were negatively correlated to sex, including LIX/CXCL5 and IL1α. When assessing correlations to genotype, we identified moderate correlations to estradiol, IL6, IL17, KC/CXCL1, and MMP8. Genotype was negatively correlated to Rantes/CCL5, VEGFα, and IL13.

When identifying if there were correlations between the serum markers, a marker of cardiovascular disease, MCP1, generated the most correlative relationships, followed closely by the inflammatory marker IL5. Not only was MCP1 correlated to the presence of plaque in both parts of the aorta, but also to G-CSF, IL4, IL5, MIP-1α/CCL3, MMP2, and MMP8.

### 3.4. Adiponectin Deficiency Promotes Atherosclerosis in Both Sexes during Aging

**Figure 4:**
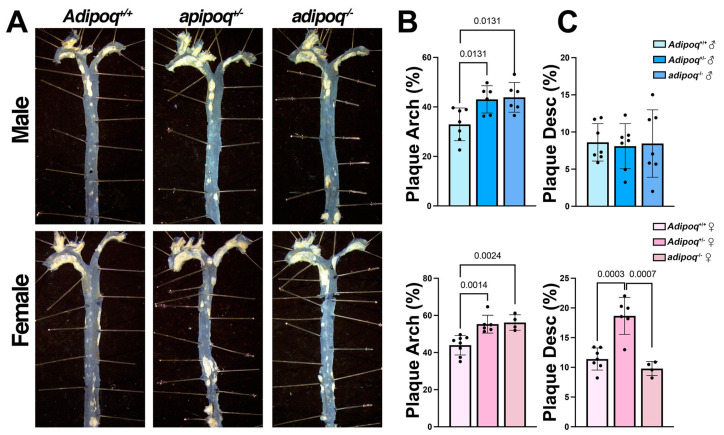
Age impairs the protective effect of adiponectin deficiency in male mice. *Apoe^−/−^Adipoq^+/+^*, *apoe^−/−^adipoq^+/−^*, and *apoe^−/−^adipoq^−/−^* male and female mice were aged to 1 year, and aortas were isolated for en face analysis of plaque (**A**). Plaque area was measured in the arch (**B**) and descending aorta (**C**) using ImageJ.

**Figure 5:**
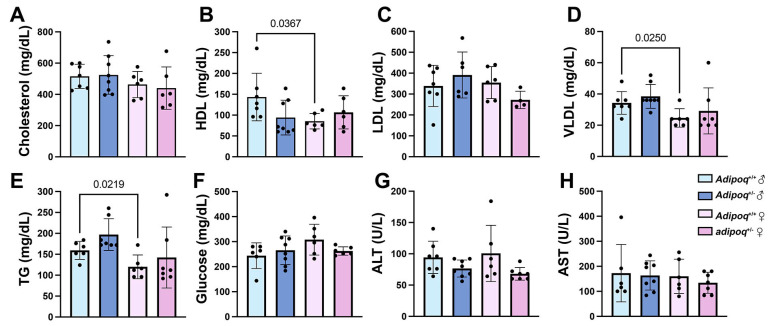
Lipid profile and glucose do not explain changes in plaque in old, adiponectin-deficient mice. Serums from one-year-old male and female *apoe*^−/−^ and *apoe*^−/−^*adipo^+/^*^−^ mice were tested for cholesterol (**A**), HDL (**B**), LDL (**C**), VLDL (**D**), TG (**E**), glucose (**F**), ALT (**G**) and AST (**H**) using a lipid plus panel and a Piccolo express analyzer (Abbott, Orlando, FL, USA).

Overall, the lack of adiponectin had a protective effect in HFD-induced plaque in adult (3–4-month-old) males, but not in female mice. We next investigated the role of aging in this genotype-dependent phenotype. The adaptation of young males to atherosclerosis was not seen in 1-year-old animals maintained on a standard chow diet, as both males and females of both genotypes exhibited higher plaque content in the arch compared with respective *apoe^−/−^* controls (Figure 4A,B). Only aged female *apoe^−/−^adipoq^+/−^* mice showed more plaque in the descending aorta (Figure 4A,C). Like animals on HFD, plaque was not associated with a worsening lipid profile or glucose levels. For this analysis, we focused on heterozygote mice because the major differences in plaque were seen in the descending aorta of female *apoe^−/−^adipoq^+/−^* mice. Cholesterol (Figure 5A), LDL (Figure 5C), VLDL (Figure 5D), and glucose (Figure 5F) were similar between genotypes in males and females. Triglycerides (TG) trended upwards, and HDL trended downwards in *apoe^−/−^adipoq^+/−^* males (Figure 5E,B, respectively). No differences were seen for females for these lipoproteins. Sex-specific differences were seen for HDL, VLDL, and TG, for which the levels were lower in females compared with male *apoe^−/−^* controls.

**Figure 6:**
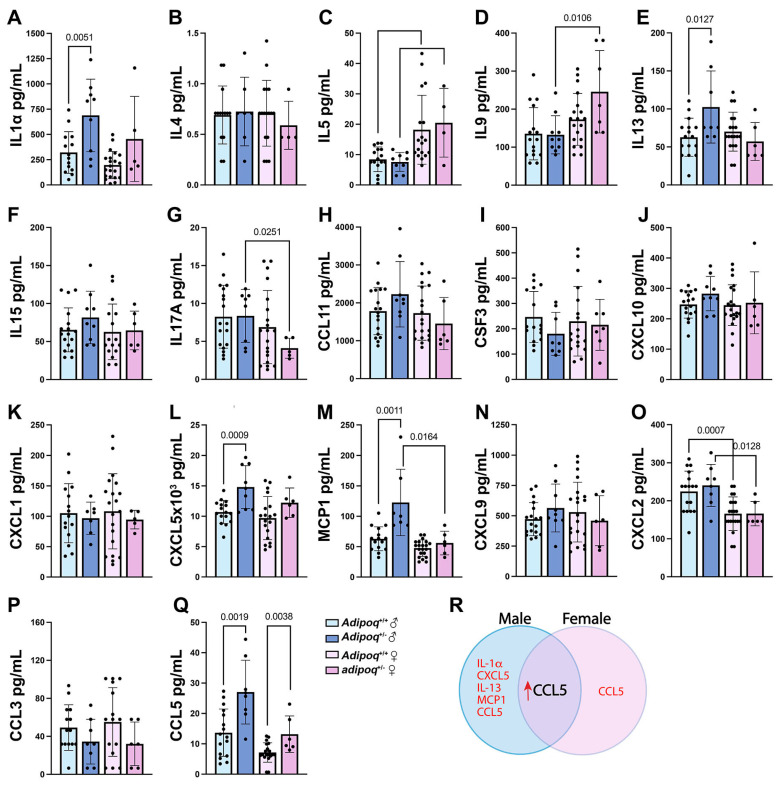
Inflammatory markers and chemokines are influenced by adiponectin and sex during aging. Serum samples from one-year-old male *apoe^−/−^* (*n* = 15–18) mice and *apoe^−/−^adipo^+/−^* (*n* = 8–9) mice and female *apoe^−/−^* (*n* = 19–21) mice and *apoe^−/−^adipo^+/−^* (*n* = 4–6) mice were tested for the expression of inflammatory markers and chemokines using the Milliplex mouse cytokine/chemokine panel 1 (**A**–**Q**). Overall changes are summarized in (**R**).

In terms of inflammatory markers, the major changes were seen in male *apoe^−/−^adipoq^+/−^* mice, who showed increases in IL1α (Figure 6A), IL13 (Figure 6E), CXCL5 (Figure 6L), and MCP1 (Figure 6M). The only molecule upregulated in both male and female adipoq^+/−^ mice was CCL5 (Figure 6Q), and IL1α was the only cytokine that was also upregulated in knockout males. In contrast to mice fed HFD, no downregulation was seen in inflammatory molecules except for CCL3, which was reduced in *apoe^−/−^adipoq^−/−^* males.

## 4. Discussion

Although adiponectin has many protective effects on the cardiovascular system, there have been discrepancies in the literature concerning its effect on atherosclerosis. Nawrocki et al. reported that adiponectin deficiency did not affect atherosclerosis in male and female *ldlr*^−/−^*adipoq*^−/−^ mice [8]. The study also used male *apoe*^−/−^*adipoq*^−/−^ mice that showed less plaque compared with *apoe*^−/−^ mice, although differences were not statistically significant. Lindgren et al., who knocked out AR2 and ApoE, reported that lack of AR2 reduced plaque accumulation in the brachiocephalic artery, but not in the arch or descending aorta of male mice [13]. Fujishima et al. reported that a deficiency in adiponectin or T-cadherin in *apoe*^−/−^ mice promoted plaque accumulation in the descending aorta [8]. Adiponectin supplementation in *apoe*^−/−^ mice, on the other hand, showed many protective effects, including inhibition of lesion formation in the aortic sinus [7,14,15], reduced inflammation caused by NF-κB [14], increased superoxide dismutase (SOD) activity, and decreased cholesterol and triglycerides [15]. These studies were performed in young mice on HFD; however, the role of adiponectin deficiency during aging is unknown.

In contrast to previous work, our study revealed novel genotype-dependent effects that differed by sex and potentially age-specific effects of adiponectin deficiency (heterozygote) in atherosclerosis in *apoe*^−/−^ mice. HFD reduced plaque in young males and increased it in young females. However, in older mice maintained on a standard chow diet, both sexes showed increased plaque compared with *apoe*^−/−^ controls. To further understand if aging affects HFD-induced plaque accumulation through different mechanisms compared to the young animals maintained on HFD, future studies on aged mice are required. Our findings in young female aortas with total and partial adiponectin deficiency are particularly important because there has only been one other study performed measuring plaque in female *apoe*^−/−^*adipoq*^−/−^ mice [3]. Additionally, no studies of plaque accumulation have been conducted in female or male *apoe*^−/−^*adipoq^+/^*^−^ mice. Although our study was consistent with Lindgren et al.’s report showing that lack of adiponectin signaling protects against atherosclerosis, this study differs because we show differences in plaque in the arch and descending aorta rather than the brachiocephalic artery. Because only males were investigated by Lindgren et al., it is unknown whether the lack of AR2 would also increase plaque in females. Several other differences in study design between these reports could also explain the differential outcomes. For example, Nawrocki et al. treated 8-week-old animals for 12 weeks with Paigen’s atherogenic diet (35% kcal from fat, 45% kcal from carbohydrates, 1.25% cholesterol, and 0.5% cholic acid), Lindgren et al. treated 8-week-old animals for 14 weeks with a lard diet (21% swine lard and 0.15% cholesterol), and Fujishima et al. treated 8-week-old animals for 12 weeks with HFD (20% fat and 0.15% cholesterol) [16]. In the present study, we fed animals with Paigen’s atherogenic diet for only 5 weeks. The short treatment time of our study likely allowed male mice to mount a compensatory mechanism that was not observed in females.

Interestingly, Fujishima et al. showed that T-cadherin mediates the binding of adiponectin to the vascular wall, as the lack of T-cadherin reduced adiponectin expression in the vasculature, thus increasing the levels of this adipokine in circulation [16]. T-cadherin has two ligands—adiponectin and LDL—that have opposite effects in atherosclerosis. T-cadherin binds to the high-molecular-weight form of adiponectin, which is the most active [17]. Low levels of adiponectin increase the availability of T-cadherin binding to LDL, thus leading to pro-atherogenic effects. Our data suggest that sex is a variable in the effects of adiponectin, as the lack of adiponectin was protective in young males, but not in females.

The effect of adiponectin deficiency on plaque accumulation induced by HFD was independent of circulating cholesterol but was associated with a reduction in MCP1, TNFα, IL4, and CXCL10 in males and an increase in MCP1, IL6, and CXCL1 in females. These cytokines have major roles in plaque formation; for example, MCP1 mediates the recruitment of monocytes to injury sites *in* the vasculature and was shown to promote plaque instability in humans [18]. In mice, genetic ablation of MCP1 protects from atherosclerosis [19]. IL4 induces MCP1 expression through a redox-dependent mechanism [20], suggesting that reduced IL4 could be the cause of MCP1 downregulation in males. CXCL10, on the other hand, promotes atherosclerosis by regulating the ratio of effector and regulatory T cells in plaques [20]. CSF3, a growth factor involved in the production of mature neutrophils, was upregulated in males and reduced in females by adiponectin deficiency. A meta-analysis suggests that CSF3 administration reduces atherosclerosis, likely by mobilizing progenitor cells [21]. Correlation analysis showed that MCP1 followed by IL5 levels are strongly correlated with plaque accumulation. The mechanism by which adiponectin regulates MCP1 and IL5 has yet to be determined. Interestingly, MCP1 was upregulated in old adiponectin heterozygote males, but not in females, suggesting that MCP1 is a major regulator of atherosclerosis in adiponectin-deficient males. As mentioned previously, treatment of old mice with HFD is needed to compare the effect of MCP1 in HFD-induced plaque accumulation during aging. However, aging as an independent factor also shows a sex-specific effect on MCP1 expression. These data also suggest that the complete knockout of adiponectin modulates atherosclerosis through a different mechanism during aging.

CCL5 was the only pro-atherogenic molecule upregulated in old male and female adiponectin heterozygotes. This cytokine has been associated with early stages of plaque formation through the recruitment of monocytes and macrophages to the lesion site [22]. Furthermore, expression of CCL5 in VSMCs was associated with plaque formation in *apoe*^−/−^ mice [23].

Additionally, we identified a strong correlation between sex and plaque area in the arch, but not in the descending aorta, and between sex and G-CSF, IL5, MIP1α/CCL3, and MMPP2. Interestingly, we recently demonstrated differential expression of MMP2 and MMP3 in the aorta of male and female C57Bl/6 wild type (WT) and *adipoq^−/−^* mice [24]. MMP2 and MMP3 were upregulated in adiponectin-deficient males compared with WT, while MMP3 and decorin were upregulated in female knockouts. It remains to be determined whether the expression profile of MMPs and extracellular matrix proteins are also regulated by sex and adiponectin in the aorta in the *apoe^−/−^adipoq^−/−^* mice.

The strong effect seen for plaque area and inflammatory cytokines in heterozygotes is intriguing. Our data suggest that a reduction in adiponectin expression, as seen in diabetes and obesity, is sufficient to promote a pro-atherogenic environment in the aorta and circulation.

Overall, we identified protective pathways that may explain the reduced plaque accumulation in young males with adiponectin deficiency. In vivo, these mechanisms include decreases in inflammatory markers, including MCP1, TNFα, IL4, and CXCL10, CSF3 and estradiol upregulations. Our data suggest that a reduction of about 50% in adiponectin promotes inflammation and atherosclerosis during aging.

### Limitations

Limitations in this study included the lack of serum samples from aging *apoe*^−/−^*adipoq*^−/−^ males and females to analyze blood lipid profiles and inflammatory and cardiovascular disease markers. Our study design concerning the choice of diet and genotype of animals provided a novel outcome by using the heterogenous adiponectin males and females and using both sexes for each genotype for each diet. However, one limitation that arose from this choice was the inability to compare our data to other research studies because the heterozygote animals have not been studied previously. We also see the value in assessing the impact of HFD on aged adiponectin-deficient and heterogenous adiponectin animals; while we were unable to include this group in the current study, this is a future research direction we will pursue. Additionally, while we show differences in plaque area, it is unknown whether plaque composition/stability was also regulated by adiponectin. For instance, adiponectin may alter plaque composition and fibrosis by regulating the extracellular matrix. Future studies are required to assess the role of adiponectin in plaque composition in response to diet and aging. Another limitation is that only cholesterol was measured in samples from HFD-treated mice because the high-fat content of the samples interfered with the analysis of other lipoproteins. Further analysis of plaque and aorta samples is also required thanks to the plethora of information gained from our serum analyses. However, due to limited protein samples and due to the nature of the en face analysis, we were unable to save plaques for macrophage, SMC, collagen, necrotic core, and MMP status.

## Figures and Tables

**Table 1:** Correlation between plaque area and pro- and anti-atherogenic markers.

Variable	*n*	Mean	SD	% Plaque Area (arch)	% Plaque Area (desc)
CHOL	33	131.36	52.26	−0.291	0.192
ALT	33	22.30	17.91	−0.238	−0.206
AST	33	37.67	28.23	0.203	0.159
Glu	29	27.41	11.31	−0.166	0.115
G-CSF	29	968.37	839.28	0.331	0.074
Eotaxin	32	2212.93	626.40	−0.126	0.033
estradiol	26	0.22	0.20	0.349	−0.152
IL1α	29	507.76	516.39	−0.452 *	−0.27
IL2	24	3.34	1.54	0.091	0.073
IL4	29	0.78	0.33	0.362	0.498 **
IL5	28	15.14	11.65	0.391 *	0.108
IL6	27	28.63	23.94	0.159	0.225
IL9	24	99.94	30.17	−0.348	0.156
IL13	29	91.65	27.85	−0.075	0.133
IL17	30	5.46	4.31	−0.497 **	−0.121
IP-10/CXCL10	29	361.60	39.98	0.326	0.265
KC/CXCL1	30	199.51	94.44	0.237	0.049
LIX/CXCL5	32	14,464.27	8165.76	−0.303	−0.349
MCP1	27	188.14	107.77	0.615 **	0.426 *
MIP1α/CCL3	30	102.33	50.15	0.287	0.218
MIP2/CXCL2	29	158.25	36.42	−0.035	−0.064
MIG/CXCL9	32	656.68	166.38	−0.134	0.048
MMP2	31	344,472.71	119,686.43	0.678 **	0.224
MMP3	32	29,474.39	14,747.21	0.134	−0.054
MMP8	30	97,594.46	41,997.43	0.207	−0.086
Rantes/CCL5	31	23.13	14.82	0.004	−0.005
TNFα	29	11.80	4.42	−0.291	0.192
VEGFα	28	0.75	0.51	−0.276	−0.205

** indicates *p* < 0.01. * indicates *p* < 0.05.

## Data Availability

All data is presented in this manuscript. Data is not stored in repositories or any other source.

## Supplementary Materials

The following supporting information can be downloaded at: https://www.mdpi.com/article/10.3390/cells13010001/s1, Figure S1: Body weight, food, and water intake in HFD-treated mice; Figure S2: Correlation analysis of plaque with sex, age and pro-atherogenic molecules.
