# Supplementary material for: The Duality of Adiponectin: The Role of Sex in Atherosclerosis
Source: Cells. 2023 Dec 19;13(1):1. doi: 10.3390/cells13010001 (PMC10778560; doi:10.3390/cells13010001)
Supplement: Supplementary file 1 [file cells-13-00001-s001.zip › cells-2777017-supplementary.pptx]

## Slide 1
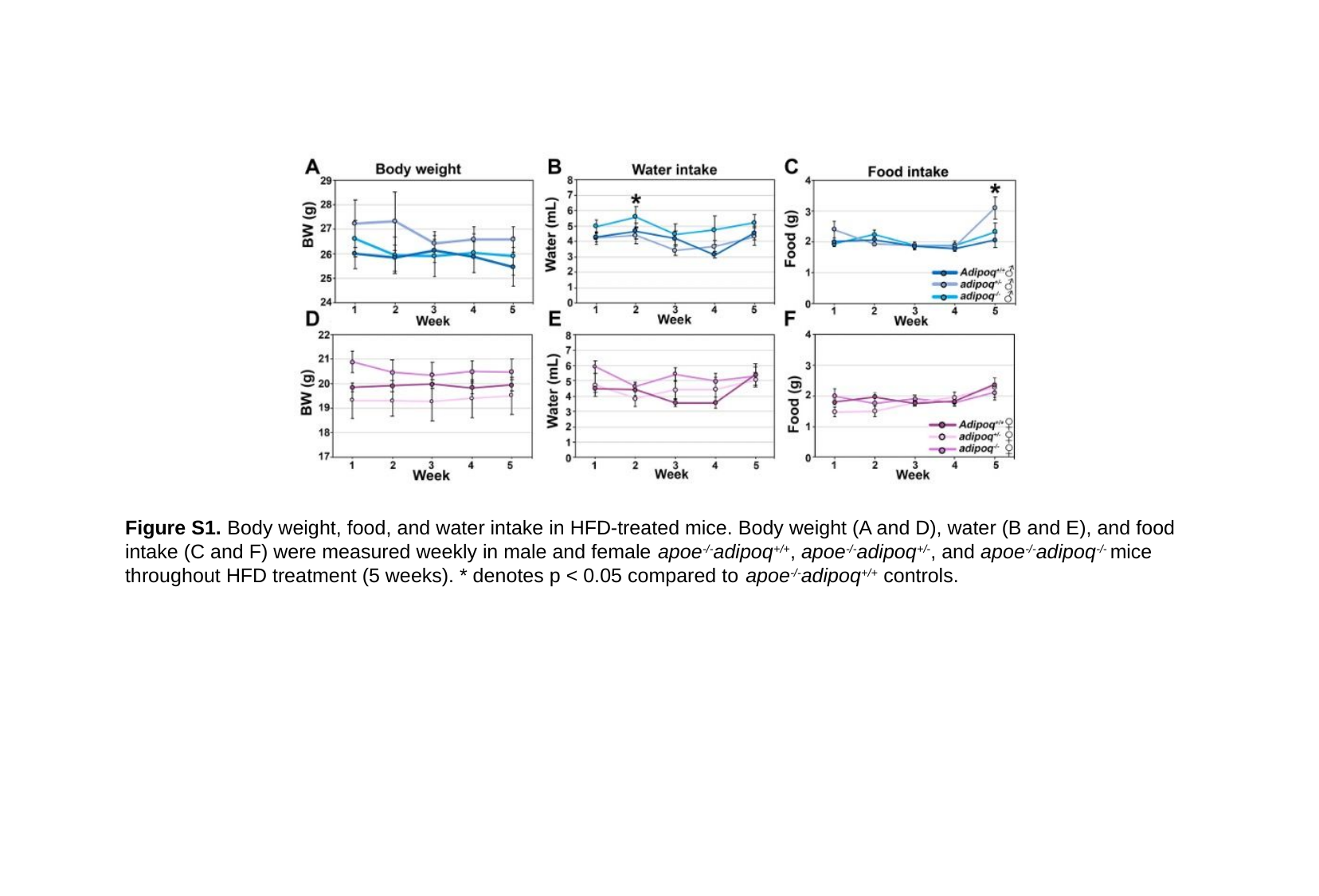

Figure S1. Body weight, food, and water intake in HFD-treated mice. Body weight (A and D), water (B and E), and food intake (C and F) were measured weekly in male and female apoe-/-adipoq+/+, apoe-/-adipoq+/-, and apoe-/-adipoq-/- mice throughout HFD treatment (5 weeks). * denotes p < 0.05 compared to apoe-/-adipoq+/+ controls.

## Slide 2
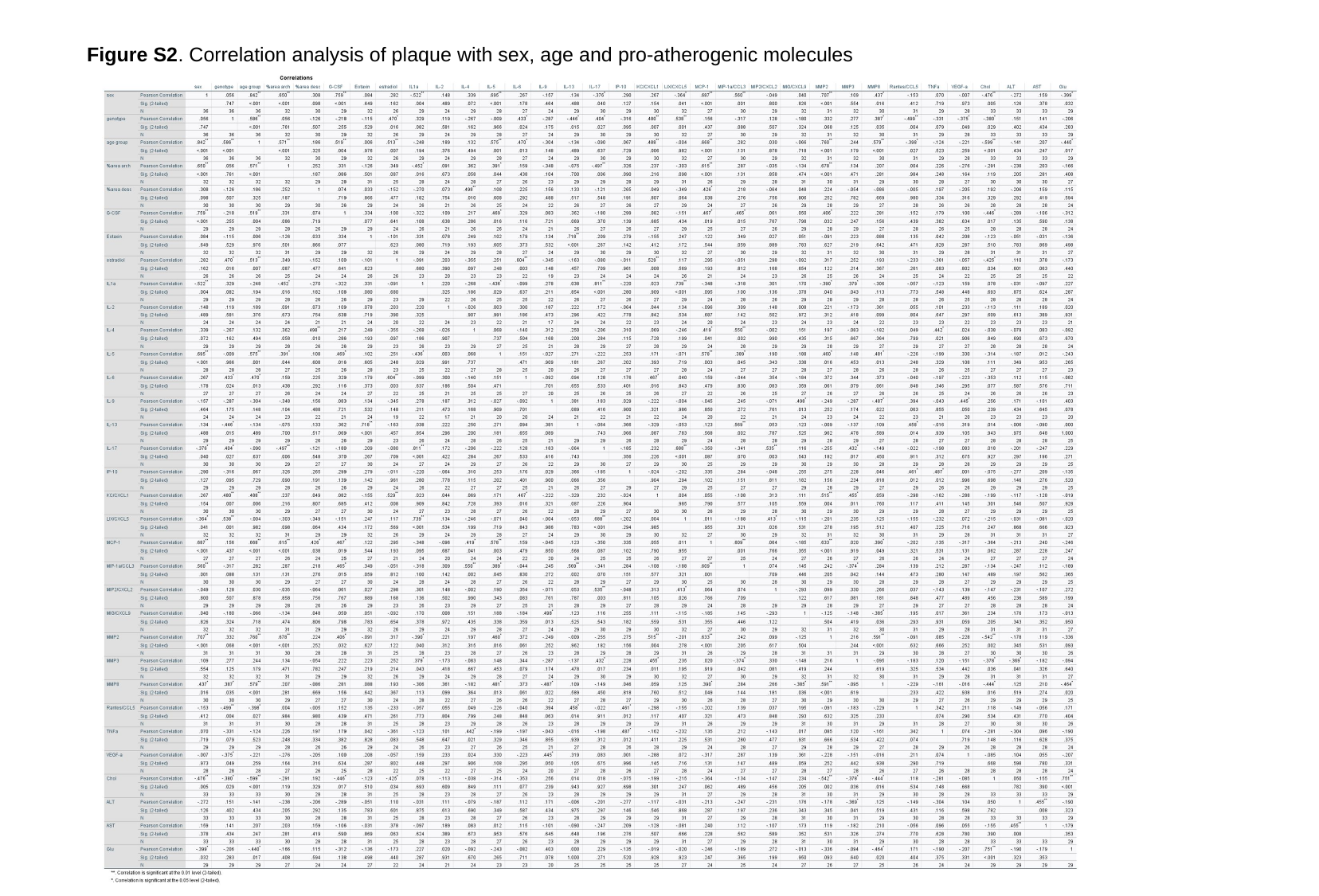

Figure S2. Correlation analysis of plaque with sex, age and pro-atherogenic molecules
